# Elucidating the molecular mechanisms of Daifu decoction in ulcerative colitis treatment through a multi-omics framework and experimental verification

**DOI:** 10.3389/fimmu.2026.1780747

**Published:** 2026-03-24

**Authors:** Yangyang Zhao, Danyang Cui, Yang Gong, Yanan Xiao

**Affiliations:** Department of Traditional Chinese Medicine, General Hospital of Northern Theater Command, Shenyang, Liaoning, China

**Keywords:** Daifu decoction, gut microbiota, multi-omics, PRKCG, ulcerative colitis

## Abstract

**Background:**

Ulcerative colitis (UC) is a significant and challenging condition in the digestive system, necessitating the development of effective therapeutic interventions. The Daifu decoction (DFD) is derived from the Lizhong decoction, a classic traditional Chinese Medicine (TCM) formula used to treat digestive diseases. Previous studies have found that DFD has a clear therapeutic effect on UC. This study aimed to investigate the underlying mechanisms of DFD in UC treatment.

**Methods:**

A mouse model of dextran sulfate sodium (DSS)-induced UC was established, and mice were treated with DFD using the concentrations 1.6 g/(kg·d), 3.2 g/(kg·d), and 6.4 g/(kg·d) for 7 days. H&E and AB-PAS staining, ELISA, qPCR, and immunofluorescence were selected to assess the role of DFD in alleviating colitis and improving intestinal barrier damage. In addition, untargeted metabolomics and transcriptomics helped investigate the potential mechanism of DFD against UC, 16S rRNA sequencing evaluated the characteristics of the gut microbiota. The key target and downstream molecules were verified by molecular docking, molecular dynamics simulation, qPCR, western blotting, and immunohistochemistry.

**Results:**

DFD prevented goblet cell loss, downregulated proinflammatory factors in serum, and improved tight junction mRNA and protein expression in colon tissue. Furthermore, DFD decreased the proportion of pathogenic microbes, while increasing the microbiota diversity and the proportion of beneficial bacterial. The integrated multi-omics analysis revealed that PRKCG was the key target of DFD to improve UC. Molecular docking and molecular dynamics simulations further confirmed that the active components of DFD could bind to PRKCG stably. The results of qPCR, western blotting, and immunohistochemistry revealed that DFD could regulate PKCγ/ERK/NF-κB pathway.

**Conclusion:**

DFD can exert therapeutic effects on UC by regulating the PKCγ/ERK/NF-κB signaling pathway and the gut microbiota to restore the intestinal barrier and decrease the secretion of proinflammatory cytokines.

## 1 Introduction

Ulcerative colitis (UC) is an inflammatory disorder that affects the rectum and colon and causes abdominal pain, diarrhea, and mucopurulent and bloody stool (1, 2). Although therapies for UC, including glucocorticoids, aminosalicylic acid, biologics, and immunosuppressants, have been verified to be favorable for attenuating symptoms (3), UC still recurs frequently, is difficult to cure completely, and even increases the risk of colon cancer due to the associated unknown etiology and pathogenesis (3–5). Approximately 0.3% of individuals worldwide have been diagnosed with UC, and the number is increasing (6), which is imposing an enormous burden on patients and society. Accordingly, exploring new effective therapies is particularly urgent.

Currently, significant progress has been made by the application of traditional Chinese medicine (TCM) in the management of UC and has shown outstanding advantages in reducing disease recurrence (7, 8). Lizhong decoction is an ancient TCM formula that was first proposed by Zhongjing Zhang, a famous physician of the Han Dynasty in China, in his book “Treatise on Febrile Diseases”. Lizhong decoction has been used to treat diarrhea for nearly 2,000 years. In recent years, in the clinical treatment of UC, Lizhong decoction has shown significant efficacy and has achieved additional benefits to patients (9). Based on many years of clinical experience in the treatment of UC and the pathogenesis evolution of UC in TCM, our research team has modified the Lizhong decoction and renamed it Daifu decoction (DFD) (China National Patent on Inventions, No.201510162437. X). In multiple clinical studies, DFD has been shown to have a clear therapeutic effect on UC and an advantage in preventing UC recurrence (10–12). In addition, we also observed that DFD had a significant repair effect on the damaged intestinal mucosal barrier in a mouse model of UC (12). Nevertheless, the mechanisms underlying the success of the DFD approach has not been fully explained. Thus, a complete understanding of the mechanism by which DFD improves UC will provide new insights into the effective treatment outcomes.

Today, multi-omics technology is one of the main methods used to elucidate the pathogenesis of diseases and to identify targets for drug treatment (13). Among them, transcriptome sequencing can be used to study differences in gene expression in disease states, whereas metabolomics can be applied to evaluate differences in metabolites in tissues and body fluids (14). The integrated analysis of the two methods can compensate for the deficiency of single omics in analyzing the complex mechanism of the disease and help to study the pathogenesis of UC and the targets of drug treatment of UC from a systemic and general perspective (15). This coincides with the principle of treating diseases with TCM.

In addition, the gut flora is closely involved in the pathogenesis of UC (16). The diversity of the gut flora in individuals with UC is reduced, manifesting itself as a change in the structure of the flora characterized by the disruption of the dynamic balance between beneficial and pernicious bacteria (17). An imbalance in intestinal flora can lead to the production of abnormal metabolites and influence gene expression, causing phenotypic changes (18). Therefore, a dysbiosis in the gut microbiota is considered one of the main mechanisms that leads to UC, and controlling this microbiome may be a useful therapeutic approach.

In this study, our objective was to explore the therapeutic mechanism of DFD in the progression of UC through a colitis model established using dextran sulfate sodium (DSS). To fully demonstrate the potential of DFD, we administered DFD to UC model mice by gavage and evaluated the effects of DFD on serum inflammatory factors, colonic histopathology, and intestinal barrier-related indices. Additionally, combining insights from transcriptomics and metabolomics, we thoroughly examined how DFD affects the management of UC. We further validated the mechanism by molecular docking, molecular dynamics simulation, Real-Time Quantitative Polymerase Chain Reaction (qPCR), immunohistochemistry (IHC), and western blotting studies. In addition, we have clarified the key role of DFD in the regulation of the balance of the intestinal flora of UC through 16S rRNA sequencing. Together, this study provides deeper insight into the therapeutic value of DFD and reveals how DFD improves UC at the level of the composition of the gut microbiota, metabolism, gene expression, and phenotype, laying a solid empirical basis for future studies on DFD, especially in terms of clinical translation and development.

## 2 Materials and methods

### 2.1 Antibodies and reagents

This study used Etiasa’s Mesalazine SR Granules (China) and DSS (SparkJade, China) with 36,000 to 50,000 molecular weights. Wuhan’s ABclonal (China) supplied mouse ELISA kits for IL-1β (RK00006), TNF-α (RK00027), and IL-6 (RK00008). Wuhan’s ABclonal (China) supplied the total RNA Extraction Reagent (TRIzol) (RK30129), 2X Universal SYBR Green Fast qPCR Mix Kit (RK21203), and ABScript Neo RT Master Mix for qPCR with the gDNA Remover reverse transcription kit (RK20433). Proteintech (Wuhan, China) provided anti-ERK1/2 (11257-1-AP) and anti-ZO-1 (21773-1-AP). Anti-claudin-1 (AB307692), anti-occludin (AB216327) were obtained from Abcam (Cambridge, MA, USA), and anti-claudin-2 (TA0128s) was obtained from Abmart (Shanghai, China). Absin (Shanghai, China) provided anti-p-PKCγ (abs148837). ABclonal (Wuhan, China) supplied anti-PKCγ (A9565), anti-p-ERK1/2 (AP0485), anti-p-NF-κB p65 (AP1294), and anti-NF-κB p65(A11202).

### 2.2 Preparation of DFD

DFD consists of the following drugs: *Aconitum carmichaelii* Debx (Fuzi, 5 g) (Lot. No. 2402001), *Zingiber officinale* Rosc. (Ganjiang, 6 g) (Lot. No. 2401002), *Cinnamomum cassia* Presl (Rougui, 6 g) (Lot. No. 99023010), *Indigo naturalis* (Qingdai, 2 g) (Lot. No. 1706001), *Sanguisorba officinalis* L. (Diyu, 10 g) (Lot. No. 22063002), *Glycyrrhiza uralensis* Fisch (Gancao, 3 g) (Lot. No. 2212005), and *Agrimonia pilosa* Ledeb (Xianhecao, 10 g) (Lot. No. 230590701). The General Hospital of Northern Theater Command’s Traditional Chinese Medicine Pharmacy prepared all of medications. Ultra-performance liquid chromatography-mass spectrometry defined the DFD fingerprint chromatogram, and the quality control procedures are described in our previous study (12). The above drugs were soaked for 60 min, then boiled in distilled water and filtered. The filtrate was finally concentrated at a concentration of approximately 0.637 g/mL and stored at 4 °C.

### 2.3 Animals

Liaoning Changsheng Biotechnology Co., Ltd. (Liaoning, China) supplied C57BL/6 male mice that weighed 20 to 22 g and were 6 to 8 weeks old. The mice were housed in the Department of Laboratory Animals of General Hospital of Northern Theater Command. This study was carried out in accordance with ARRIVE recommendations and guidelines for the care and use of laboratory animals and received approval from the Ethics Committee of the General Hospital of Northern Theater Command (No. 2022-02).

### 2.4 UC model induction and drug administration

After 1 week of adaptive feeding, the mice (n=10) were randomly assigned to normal control (NC), model (DSS), mesalazine (MES), high-dose DFD (DFD-H), middle-dose DFD (DFD-M), and low-dose DFD (DFD-L) groups. In addition to the NC group, the other groups were given a 2.5% DSS solution for 7 days to induce colitis. From Day 4, the mice in the MES and DFD groups were given mesalazine and DFD, respectively, and the other groups received distilled water of the same volume. The clinical oral dose of DFD was 42 g/d (calculated based on an adult body weight of 60 kg), corresponding to 0.7 g/(kg·d), and was transformed to an equivalent gavage dose of 6.4 g/(kg·d) (calculated based on a mouse body weight of 20 g) in the DFD-H group. The gavage dose used in the DFD-M group was 3.2 g/(kg·d), which was half of that used in the DFD-H group; the gavage dose used in the DFD-L group was 1.6 g/(kg·d), which was one-quarter of that used in the DFD-H group. The clinical oral dose of mesalazine was 4 g/day (calculated based on an adult body weight of 60 kg), which was converted to a mouse equivalent gavage dose of 0.61 g/(kg·d) (calculated based on a mouse body weight of 20 g). On day 11, 1% pentobarbital sodium solution helped sedate the mice by intraperitoneal injection (200 mg/kg). Blood samples were obtained by removing the eyeball and were centrifuged to obtain serum. Next, the colon tissue and intestinal contents were gathered for subsequent experiments.

### 2.5 Calculation of the disease activity index

The characteristics of the stool, body weight, and hematochezia were measured daily. The DAI score was computed as previously described (16).

### 2.6 Alcian blue and periodic acid-Schiff staining and hematoxylin and eosin staining

First, 4% paraformaldehyde helped fix colon samples that were dried in ethanol, covered with paraffin, and then cut into slices that had a 4 μm thickness. AB-PAS or H&E staining was carried out. The PANNORAMIC MIDI II Digital Scanner (3DHISTECH Ltd., Budapest, Hungary) helped capture the pathological images. The photos were subsequently captured and processed using the 3DHISTECH slide viewer software.

### 2.7 Enzyme-linked immunosorbent assay

ELISA was used to determine IL-1β, TNF-α, and IL-6 according to the kit instructions.

### 2.8 Real-time quantitative polymerase chain reaction

Total RNA Extraction Reagent (TRIzol) helped extract total RNA. We used the ABScript Neo RT Master Mix for qPCR with a gDNA Remover reverse transcription kit to reverse transcribe the RNA into cDNA. The 2X Universal SYBR Green Fast qPCR Mix kit helped with additional detection. The 2^-△△Ct^ technique helped to find relative mRNA levels of PRKCG, ZO-1, claudin-2, occludin, and claudin-1. Their expression was normalized to GAPDH. Shanghai’s Sangon Biotech Co., Ltd. produced the primers. **Supplementary Table 1** lists the primer sequences.

### 2.9 Immunofluorescence

Xylene helped deparaffinize 4-μm thick slices, followed by gradient ethanol dehydration, and repair of 0.01 mol/L of citrate buffer at pH 6.0. These slices underwent 10% goat serum blocking, primary antibodies (including claudin-1, ZO-1, claudin-2, and occludin), fluorescently labelled secondary antibody cultivation, and 4’,6-diamidino-2-phenylindole staining. PANNORAMIC MIDI II Digital Scanner and 3DHISTECH software helped capture photos.

### 2.10 Western blotting

Total protein was extracted from the colon tissues by grinding them in RIPA lysis buffer, and the BCA test was used to measure protein content. SDS-PAGE was subsequently performed, followed by transfer, blocking, and primary and secondary antibody incubation. The primary antibodies used for SDS-PAGE were anti-PKCγ (1:1000), anti-p-PKCγ (1:1000), anti-ERK1/2 (1:16000), anti-p-ERK1/2 (1:2000), anti-NF-κB p65 (1:1000), and anti-p-NF-κB p65 (1:20000). Finally, Image J was used to analyze the gray values of the protein bands.

### 2.11 Immunohistochemical analysis

After a series of procedures, such as deparaffinization, dehydration, antigen repair, and blocking, the sections were incubated with primary and secondary antibodies. The primary antibodies used were p-ERK1/2 (1:1000), p-PKCγ (1:100), and p-NF-κB p65 (1:1000). After incubation, DAB staining, nuclei counterstaining with hematoxylin, and neutral gum sealing were applied to these slices. Lastly, 3DHISTECH software and a PANNORAMIC MIDI II Digital Scanner was used to obtain images.

### 2.12 16S rRNA gene sequencing analysis

For the NC, DSS, and DFD-H groups (n=6), the Illumina NovaSeq 6000 sequencing system helped to evaluate the characteristics of the gut flora. We used the intestinal contents to isolate genomic DNA, which was then used as a template for PCR to amplify the V3-V4 region of the 16S rRNA gene. Magnetic beads purified the PCR products after they had been electrophoresed in 2% agarose gels. To create the library, the TruSeq^®^ DNA PCR–Free Sample Preparation Kit was used. After library qualification, sequencing was done using the Illumina NovaSeq 6000 system. The sequenced data were subjected to beta diversity analysis, clustering, species annotation, alpha diversity analysis, species abundance composition analysis, and linear discriminant analysis was coupled with effect size measurements (LEfSe) to evaluate the composition and distribution of the gut flora.

### 2.13 Transcriptomics

Total RNA was extracted from colonic tissue samples from the NC, DSS, and DFD-H groups (n=6). The Qsep400 bioanalyzer along with the Qubit 4.0 fluorometer/MD microplate reader, RNA quality was assessed. Oligo (dT) magnetic beads helped enrich mRNAs with polyA tails. The RNA was then broken up into small fragments by adding a fragmentation buffer. Six-base random hexamers helped create the first chain cDNA, with the templates being short RNA fragments. Two-stranded cDNA was then created by adding buffer, dNTPs (dATP, dTTP, dCTP, and dGTP), and DNA polymerase. Magnetic beads for DNA purification was used to purify the double-stranded cDNA. After double-stranded cDNA was purified, it was end-repaired, an A-tail was added, and binding of sequencing connectors. DNA purification magnetic beads were then added to select fragment sizes, and PCR enrichment was lastly conducted to produce the final cDNA library. Once the library’s quality was established, sequencing was carried out using the Illumina high-throughput sequencing system. Gene expression was found using fragments per kilobase of transcript per million fragments mapped. DESeq2 helped screen differentially expressed genes (DEGs) with a false discovery rate (FDR) < 0.05, as well as a |log2 fold change|≥1. The DEGs obtained were subjected to subsequent Kyoto Encyclopedia of Genes and Genomes (KEGG) enrichment analysis and additional studies.

### 2.14 Untargeted metabolomics

The metabolic characteristics of the colon sample from the NC, DSS, and DFD-H groups (n=6) were examined by tandem liquid chromatography mass spectrometry (LC–MS/MS). The frozen samples were thawed, weighed to 20 ± 1 mg, and ground into a homogenate. The mixture was then centrifuged three times again for machine analysis after adding 400 µL of an internal standard extract made of 70% methanol–water. The Waters ACQUITY Premier HSS T3 column (1.8 µm, 2.1 mm × 100 mm) was used for chromatographic separation with the following parameters: mobile phase A containing 0.1% formic acid/water; mobile phase B containing 0.1% formic acid/acetonitrile; 0.4 mL/min flow rate; 40 °C column temperature; and 4 µL injection volume. **Supplementary Table 2** shows the mobile phase gradient settings for T3 columns. An AB TripleTOF 6600 mass spectrometer helped conduct mass spectrometry. The conditions are shown in **Supplementary Table 3**. The ProteoWizard converted the raw information into mzXML format, and the XCMS program extracted peaks, performed alignment and corrected the retention time. K-Nearest Neighbors (KNN) helped to fill in the blank values, the support vector regression (SVR) approach helped rectify the peak areas; peaks in each group with >50% missing rates were filtered. A specially designed database, which combined the prediction library, public library, and metDNA techniques, helped to identify the metabolites once the chosen peaks had been adjusted. After extracting and detecting substances with a coefficient of variation (CV) <0.3 along with a comprehensive score >0.5 for the quality control (QC) samples, the metabolites were obtained by combining the negative and positive modes. The samples were subjected to principal component analysis (PCA) before the difference analysis to compare variances within as well as between groups. Orthogonal partial least squares-discriminant analysis (OPLS-DA), alongside PCA, was conducted to determine the variable importance in projection (VIP) of the metabolites. A VIP > 1 and *P* < 0.05 were used as screening conditions to identify differentially expressed metabolites (DEMs). Subsequent KEGG and other analyses were performed on the obtained DEMs.

### 2.15 Molecular docking and molecular dynamics simulation

To verify that PRKCG is a key target for DFD in the treatment of UC, we conducted molecular docking and molecular dynamics simulation. According to the method of Daina et al. (19), the components of DFD, which we previously obtained by mass spectrometry (12), were screened for high gastrointestinal absorption (GI). The screening criteria were set as GI is high and at least 3 Yes in Druglikeness. The 3D structures of DFD components and PRKCG were obtained from the Pubchem database (https://pubchem.ncbi.nlm.nih.gov/) and the Uniprot database (https://www.uniprot.org/), respectively. OpenBabel 3.1.1 software was used to optimize the structure of the DFD components. AutoDock Tools 1.5.6 was used to hydrogenate the components and PRKCG and to determine rotatable bonds. Finally, Auto Dock Vina 1.2.5 software was used for molecular docking and PyMOL 2.3.0 software was used for visualization.

Gromacs 2024.4 software was used to conduct the molecular dynamics simulation of the complex between the five components of the DFD with the highest binding energy (including 23-hydroxytormentic acid, euscaphic acid, asiatic acid, ellagic acid, and quillaic acid) and the PRKCG protein to verify the binding stability. Detailed operational procedures are provided in the **Supplementary method**.

### 2.16 Statistical analysis

All statistical analyses were performed using GraphPad Prism 10.0 and SPSS 26. No fewer than three replications were used to generate an individual data point in each of the independent experiments, and all information was reported as the mean ± standard deviation (mean ± SD). One-way analysis of variance (ANOVA) helped evaluate statistical differences between groups. The significance threshold was *P* < 0.05.

## 3 Results

### 3.1 DFD inhibited colitis symptoms in UC mice

**Figure 1:**
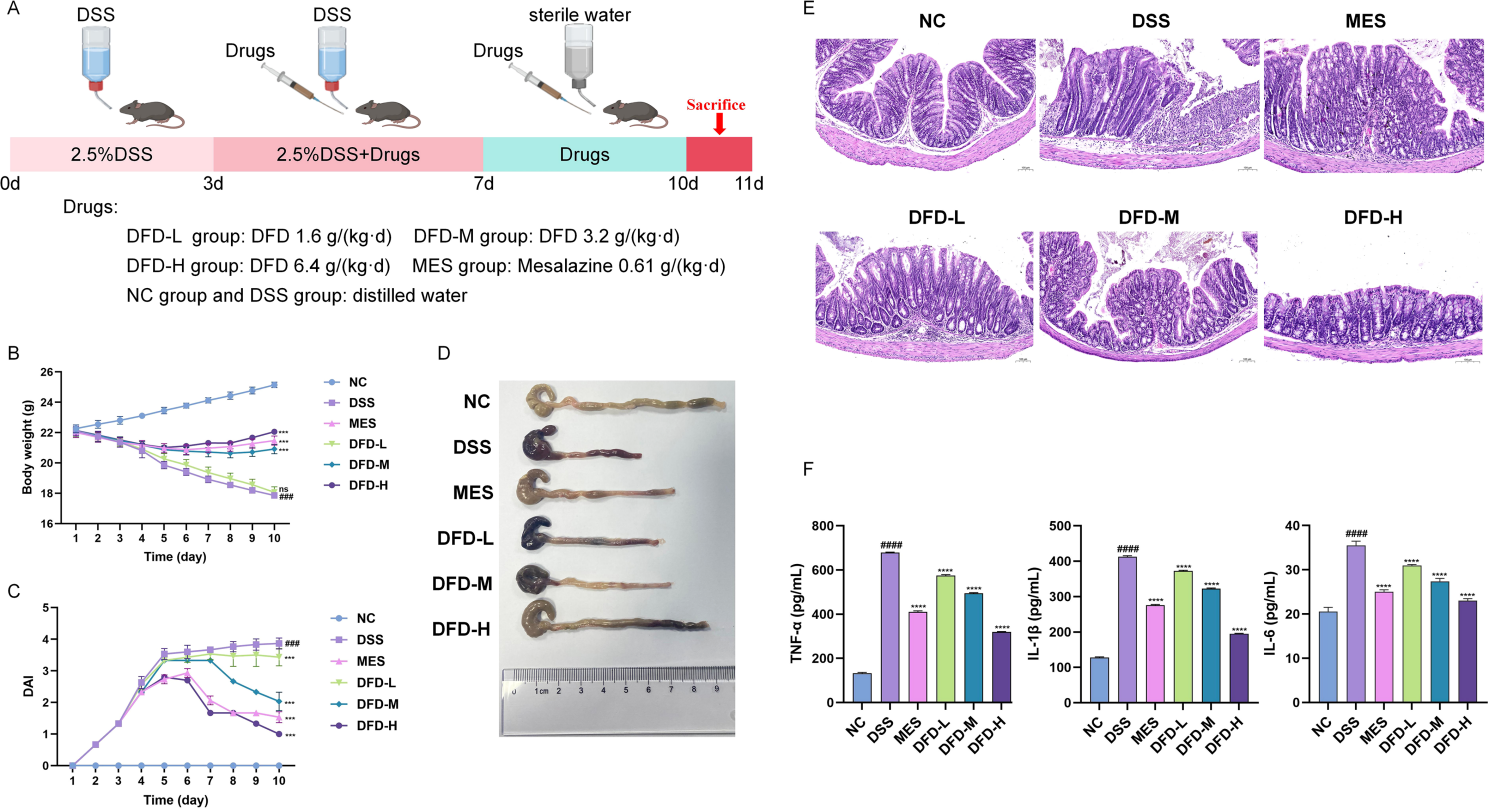
DFD alleviates DSS-induced colitis. **(A)** Schematic design of the experimental design. **(B)** Body weight. **(C)** DAI score. **(D)** Representative images of the colon. **(E)** H&E staining of colon tissue, Scale bar, 100 µm. **(F)** The levels of TNF-α, IL-1β, and IL-6 in the serum. Data are presented as mean ± SD (n = 3-10). ^###^*P* < 0.001, ^####^*P* < 0.0001 *vs*. the NC group; ^***^P < 0.001, ^****^P < 0.0001 *vs*. the DSS group; ns: not significant.

We established a DSS-induced UC model in mice; subsequently, the mice were treated with different concentrations of DFD (**Figure 1A**). As expected, DSS caused colitis manifestations in mice, including raised DAI scores, reduced colon length, and body weight loss. DFD treatment significantly attenuated the above UC manifestations (**Figures 1B–D**). Furthermore, H&E staining findings revealed that the integrity of the colonic mucosal epithelium was disrupted, the structure of the crypt was damaged, and the mucosa and submucosa were infiltrated by numerous inflammatory cells in UC model mice, whereas DFD administration effectively improved the pathological damage to the colon (**Figure 1E**). Moreover, TNF-α, IL-1β, and IL-6 levels were decreased following DFD therapy, which expanded on the curative potential DFD for the treatment of UC (**Figure 1F**). These findings suggested that DFD was a promising therapy for UC.

### 3.2 DFD enhanced the integrity of the intestinal barrier

**Figure 2:**
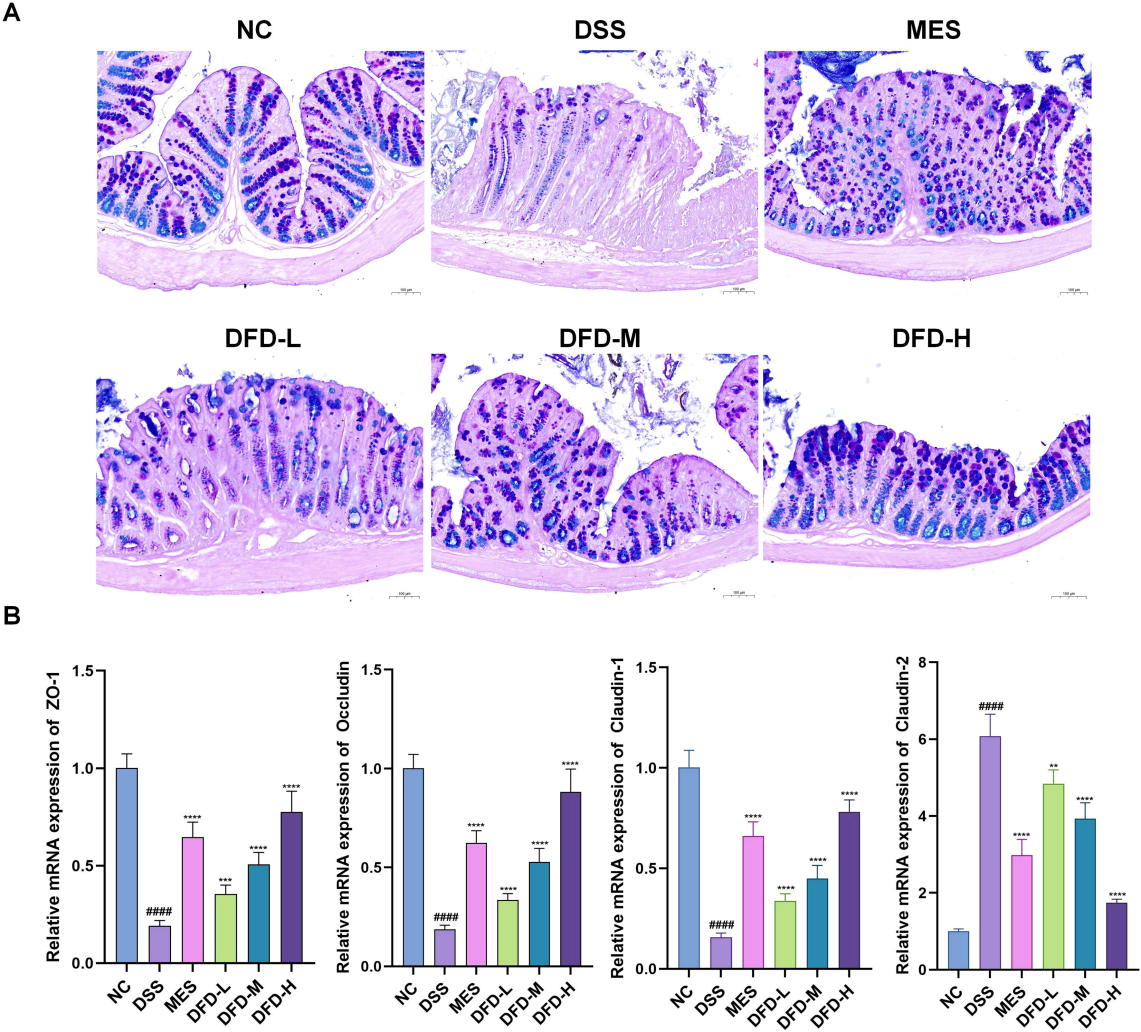
DFD alleviates intestinal barrier injury in UC model mice. **(A)** AB-PAS staining. Scale bar, 100 µm. **(B)** The mRNA levels of ZO-1, occludin, claudin-1, and claudin-2 in colon tissues. n = 3. ^####^*P* < 0.0001 *vs*. the NC group; ^**^P < 0.01, ^***^P < 0.001, ^****^P < 0.0001 *vs*. the DSS group.

We first examined the intestinal mucus barrier using AB-PAS staining to clarify the impact of DFD on the integrity of the intestinal barrier. Compared with those of normal mice, the DSS-treated mice manifested lower goblet cells numbers and mucin levels, demonstrating that the intestinal mucus barrier of the UC mice was damaged, whereas DFD treatment reduced the damage to the DSS-induced intestinal mucus barrier in the mice (**Figure 2A**). In addition, the expression of tight junction complexes was detected at the transcriptional and translational levels to clarify the changes in colonic mechanical barrier induced by the administration of DSS and DFD. qPCR revealed that claudin-1, ZO-1, and occludin mRNA levels decreased after DSS intervention, whereas claudin-2 mRNA levels were increased, and DFD administration reversed these changes (**Figure 2B**). Immunofluorescence revealed similar results at the translational level (**Supplementary Figure 1**). These results suggest that DFD ameliorates intestinal barrier damage in UC.

### 3.3 DFD regulated intestinal flora imbalance in UC mice

**Figure 3:**
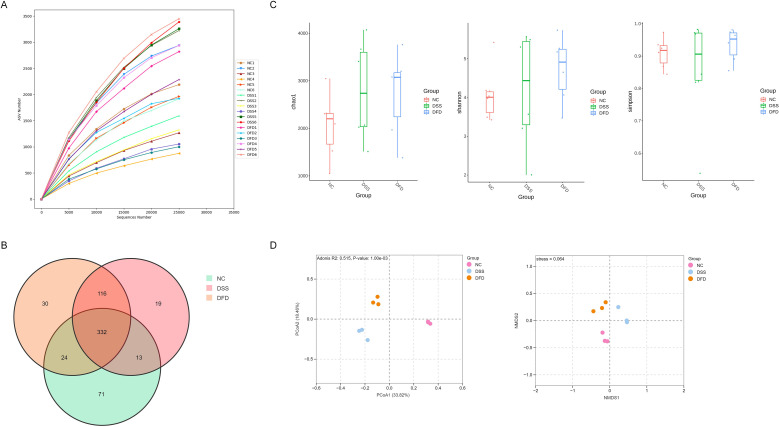
Effects of DFD on intestinal flora diversity and ASVs. **(A)** Rarefaction curve. **(B)** ASV Venn diagram. **(C)** Alpha diversity. **(D)** Beta diversity. Data are presented as mean ± SD (n = 3-6).

**Figure 4:**
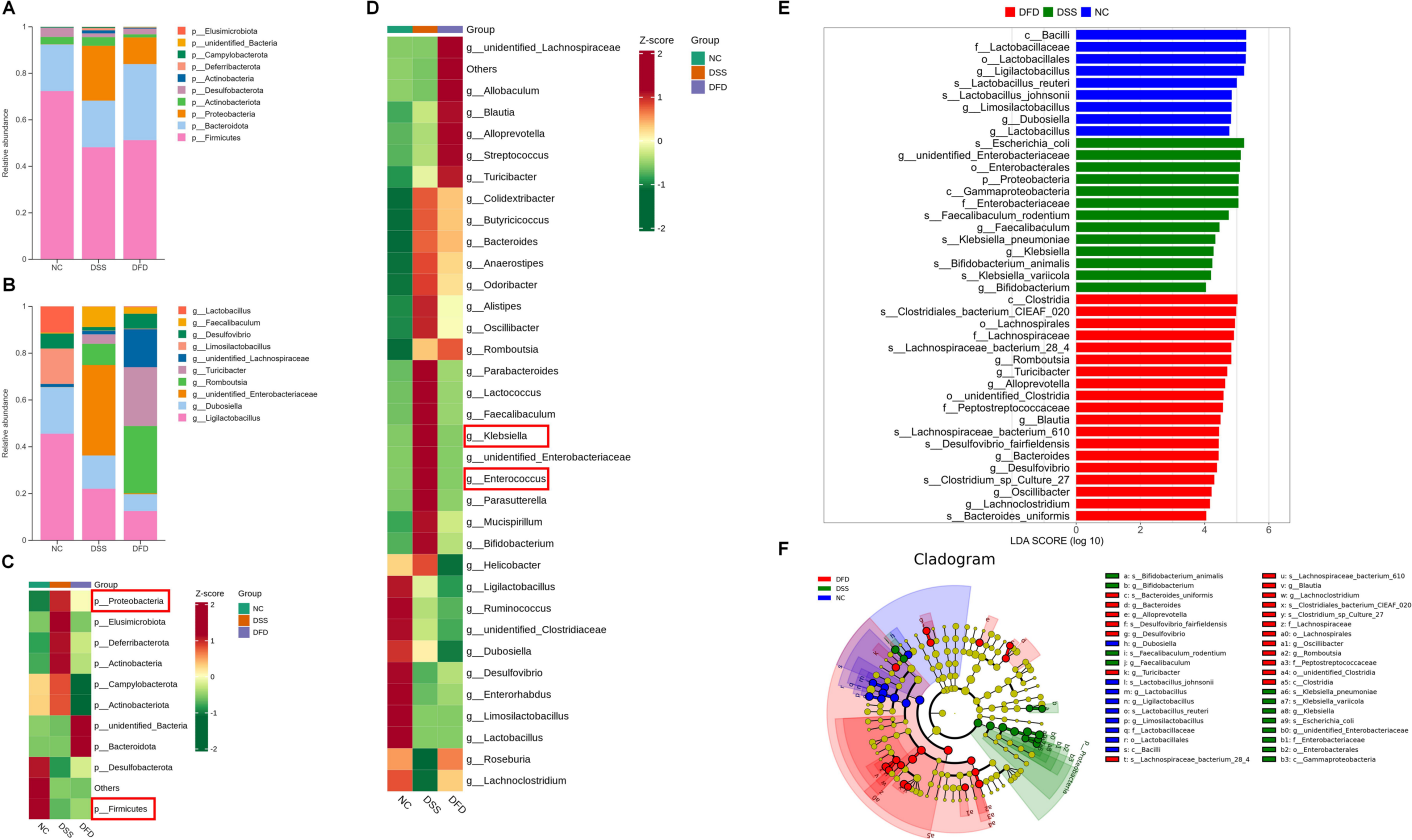
DFD regulates gut microbiota dysbiosis in UC model mice. **(A-B)** Community structure analysis at the phylum level **(A)** and genus level **(B)**. **(C, D)** Heatmap of microbiota abundance at the phylum level **(C)** and genus level **(D)**. **(E)** Histogram showing distribution with LDA scores >4. **(F)** Evolutionary branch diagram. n=6.

16S rRNA sequencing helped determine the impact of DFD on colitis at the level of the gut flora. Rarefaction curve analysis revealed that at the sequence number 25000, the majority of the gut microbial diversity was obtained, and no additional sequencing was needed (**Figure 3A**). **Figure 3B** shows the distribution of amplicon sequence variants (ASVs) in each group, a total of 332 common ASVs were identified in the three groups, and each group also their own unique ASVs. The abundance as well as variety of microbial communities in samples are reflected by alpha diversity, which helps analyze the diversity of the microbial community (20). Alpha diversity was described using the Chao1, Shannon, and Simpson indices. No apparent variance was detected in the alpha diversity amongst the three groups (**Figure 3C**). Nonetheless, the alpha diversity of the DFD group was marginally higher than that in the other groups, which may have been caused by drug usage or the experimental conditions. The shared characteristics of the microbial composition were analyzed using the beta diversity (20). Here, the beta diversity was characterized using non-metric multi-dimensional scaling (NMDS) as well as principal coordinates analysis (PCoA). Although the groups were geographically divided, microbial communities were all integrated (**Figure 3D**), suggesting the makeup of the gut flora in UC mice had been significantly altered following DFD treatment. At the genus as well as phylum levels, the taxonomic makeup of the microbiota was examined. The top 10 phylum-level microbiota in terms of abundance were *Elusimicrobiota*, *unidentified_Bacteria*, *Campylobacterota*, *Deferribacterota*, *Actinobacteria*, *Desulfobacterota*, *Actinobacteriota*, *Proteobacteria*, *Bacteroidota*, and *Firmicutes* (**Figure 4A**), among which *Firmicutes*, *Bacteroidota*, and *Proteobacteria* were the three most important phyla. DFD treatment altered levels of the three major phyla associated with DSS exposure. At the genus level, the top 10 in terms of abundance were *unidentified_Lachnospiraceae*, *Faecalibaculum*, *Desulfovibrio*, *Lactobacillus*, *Turicibacter*, *Limosilactobacillus*, *Romboutsia*, *unidentified_Enterobacteriaceae*, *Dubosiella*, and *Ligilactobacillus* (**Figure 4B**). In addition, the abundance of different groups at the genus as well as phylum levels are displayed in a heatmap (**Figures 4C,D**). DSS raised *Proteobacteria* abundance (a pathogenic bacterium) at the phylum level, but reduced *Firmicutes* abundance (a beneficial bacterium). However, this trend was reversed after treatment with DFD. DSS increased *Enterococcus* and *Klebsiella* abundance (harmful bacteria) at the genus level, and DFD reversed these variations. LEfSe was used to identify the preponderant species in the three groups, and our findings demonstrated that the preponderant flora in the colitis mice were mostly *Proteobacteria* and *Klebsiella*, which are pathogenic bacteria. However, after the DFD intervention, the preponderant flora was mainly *Turicibacter*, which is a beneficial bacterium (**Figures 4E,F**). These findings reveal that DFD can modulate DSS-induced gut microbiota dysbiosis.

### 3.4 DFD altered gene expression profiles of DSS-induced mice

**Figure 5:**
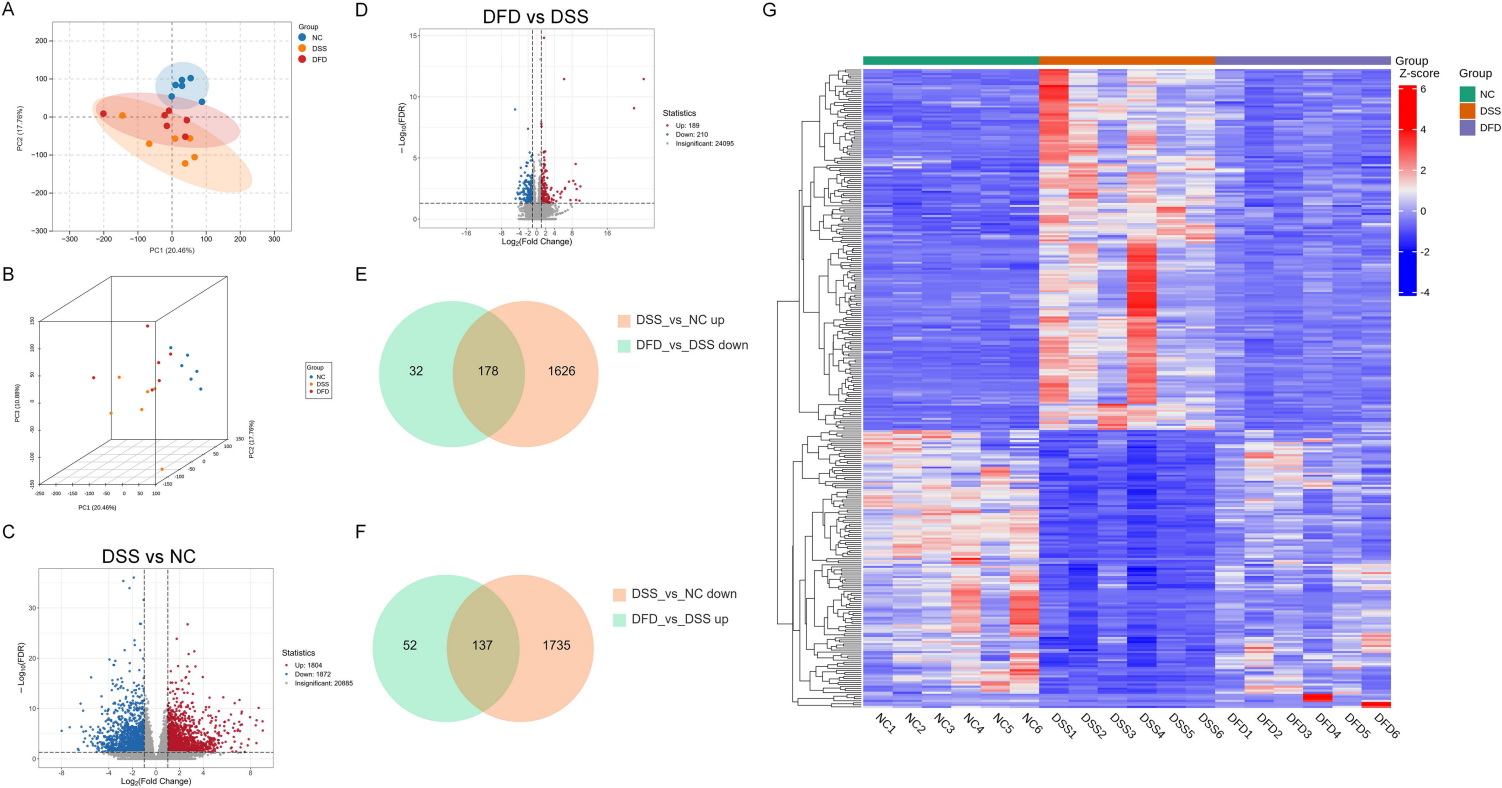
DFD alters the gene expression profile of UC mice. **(A)** 2D PCA plot. **(B)** 3D PCA plot. **(C, D)** Volcano map of the DEGs between the DSS group and the NC group **(C)** and between the DFD group and the DSS group **(D)**. **(E, F)** Venn diagrams of the DEGs in the DSS group *vs*. the NC group and the DFD group *vs*. the DSS group. **(G)** Heatmap of 315 DEGs. n = 6.

Transcriptome analysis revealed the impact of DFD on DSS-induced gene expression patterns in mice. Both 2D and 3D PCA analysis revealed that samples were well stratified between groups, the samples were well clustered within groups, and the DFD group samples tended to be closer to the NC group samples (**Figures 5A, B**), indicating that DFD treatment effectively ameliorated abnormal expression patterns associated with UC pathogenesis. Specifically, DSS induction resulted in 1,872 downregulated DEGs and the upregulation of 1,804 DEGs (**Figure 5C**). In addition, 189 DEGs were upregulated and 210 DEGs were downregulated after DFD intervention (**Figure 5D**). **Figures 5E,F** shows that the levels of 315 DEGs were altered from the disease state to the normal level following DFD intervention, of which 178 DEGs were downregulated and 137 DEGs were upregulated after DFD intervention. In addition, in **Figure 5G**, the heatmap of 315 intersecting DEGs showed that DFD intervention caused the gene expression profile to become more similar to that of a normal level. The above analysis showed that DFD treatment can effectively ameliorate abnormal gene expression patterns associated with UC pathogenesis.

### 3.5 DFD altered the metabolic characteristics of UC model mice

**Figure 6:**
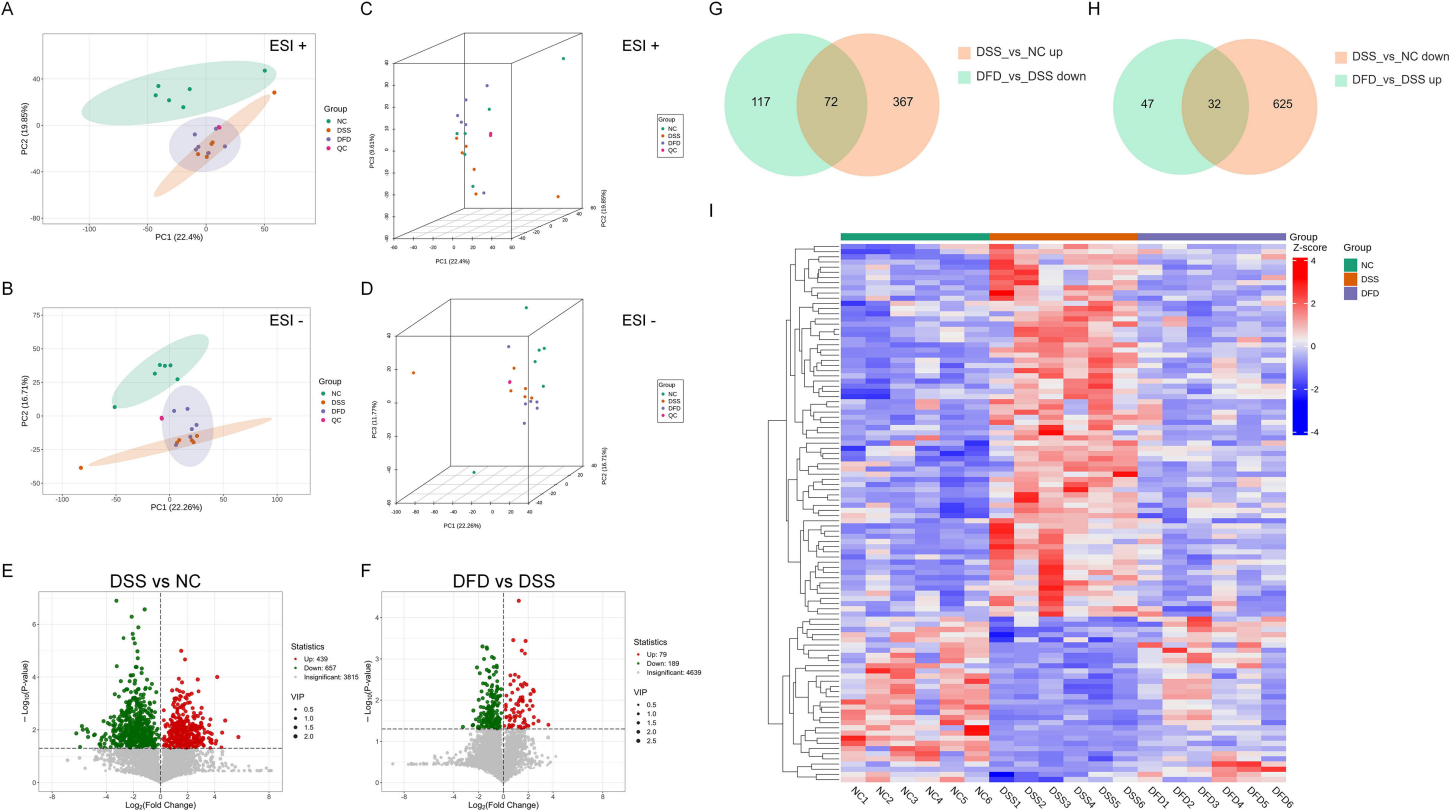
DFD alters the metabolic characteristics of UC mice. **(A)** 2D PCA plot (positive ion mode). **(B)** 2D PCA plot (negative ion mode). **(C)** 3D PCA plot (positive ion mode). **(D)** 3D PCA plot (negative ion mode). **(E, F)** Volcano maps of the DEMs in the DSS group *vs*. the NC group **(E)** and the DFD group *vs*. the DSS group **(F)**. **(G, H)** Venn diagrams of the DEMs in the DSS group *vs*. the NC group and the DFD group *vs*. the DSS group. **(I)** Heatmap of 104 DEMs. n = 6.

Colon tissue metabolomics analysis revealed the impact of DFD on the metabolic profile of mice. Using 2D as well as 3D score plots from PCA analysis, metabolic patterns of colon tissues were examined from the NC, DSS, and DFD groups. The NC and DSS groups were distinctly divided, indicating the high reliability of the model. Moreover, the DFD group tended to approach the NC group more closely; hence, DFD considerably augmented the metabolite expression profile linked to UC pathogenesis (**Figures 6A–D**). The volcano plot revealed that DSS downregulated 657 DEMs but upregulated 439 DEMs (**Figure 6E**). Additionally, 79 DEMs were upregulated and 189 DEMs were downregulated after administering DFD (**Figure 6F**). Furthermore, the Venn map revealed that 72 DEMs were upregulated after UC model establishment, whereas exposure to DFD resulted in their downregulation. In contrast, 32 DEMs were downregulated in the colitis model mice but were upregulated after DFD treatment (**Figures 6G,H**). The heatmap of 104 intersecting DEMs also showed that DFD treatment had the opposite effect on the levels of the 104 intersecting DEMs compared with the DSS group. After DFD treatment, changes of metabolites levels in colitis model mice were similar to those observed in normal mice (**Figure 6I**), indicating that the metabolic characteristics of the DFD intervention tended to be normal. Based on the above analysis, it can be concluded that DFD treatment effectively alleviated metabolic abnormalities in UC model mice.

### 3.6 Integrated analysis of multi-omics

**Figure 7:**
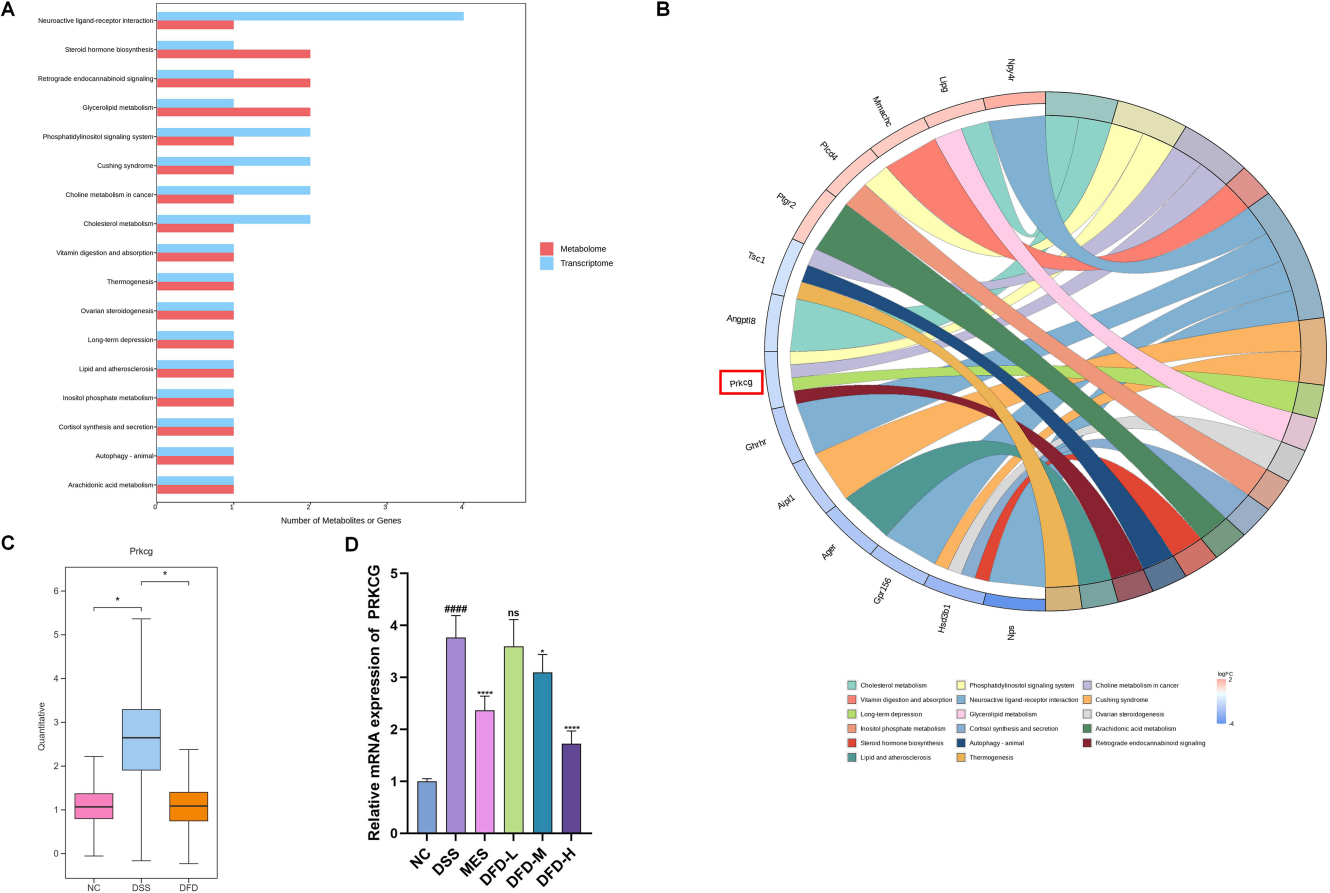
Multi-omics integrated analysis. **(A)** Intersection pathway map of DEGs and DEMs. **(B)** Chord diagram. **(C)** Relative expression of Prkcg according to colon tissue transcriptome analysis. **(D)** The level of PRKCG mRNA in colon tissue was detected by qPCR. Data are presented as mean ± SD (n = 6). ^*^P < 0.05, ^####^*P* < 0.0001 *vs*. the NC group; ^*^P < 0.05, ^****^P < 0.0001 *vs*. the DSS group; ns: not significant.

To comprehensively analyze the internal relationships between the transcriptomic and metabolomic datasets at different levels of biology, Pearson’s correlation analysis was performed on 315 DEGs and 104 DEMs. The screening conditions were as follows: the correlation filtering threshold was 0.8, and *P* < 0.05. The 249 DEGs were found to be correlated with 76 DEMs; detailed information is shown in **Supplementary Table 4**. Combined pathway enrichment analysis identified 17 pathways associated with these DEGs or DEMs (**Figure 7A**). Notably, Prkcg, a DEG, was enriched in a greater number of pathways (**Figure 7B**), indicating that changes in Prkcg expression following DFD treatment of UC, participates in multiple pathways. Boxplots revealed that DSS increased the relative content of Prkcg; however, this trend was reversed following DFD treatment (**Figure 7C**). Using qPCR to verify PRKCG mRNA levels in colon tissues, the results confirmed that DSS significantly increased PRKCG mRNA levels, whereas DFD treatment decreased these levels (**Figure 7D**), which validated the results of the transcriptomic analysis. These results provide potential molecular evidence indicating that PRKCG is a key target for DFD treating UC.

### 3.7 Results of molecular docking and molecular dynamics simulation

**Figure 8:**
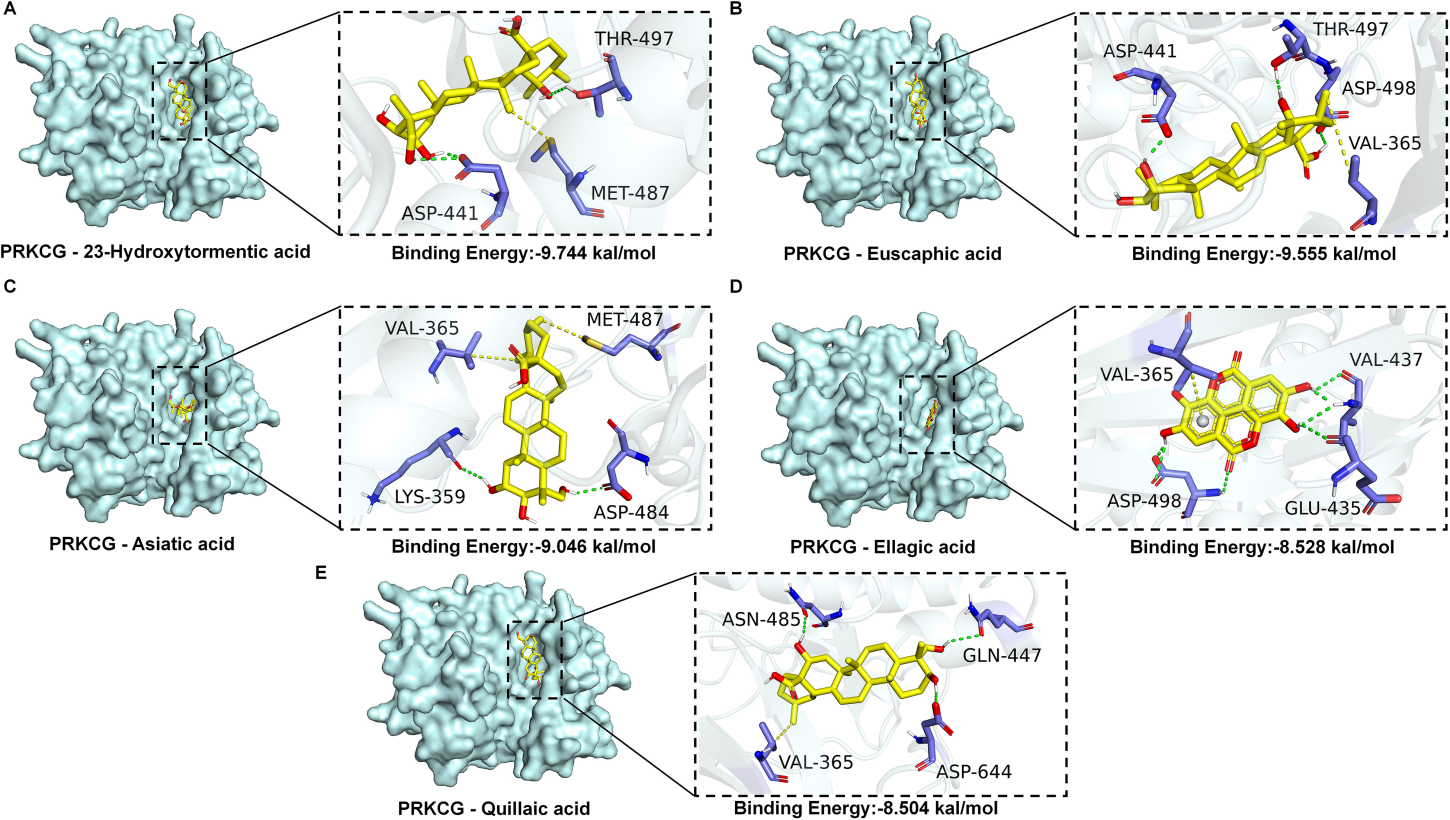
The results of molecular docking studies. **(A)** PRKCG-23-Hydroxytormentic acid. **(B)** PRKCG-Euscaphic acid. **(C)** PRKCG-Asiatic acid. **(D)** PRKCG-Ellagic acid. **(E)** PRKCG-Quillaic acid.

To verify the regulatory effects of DFD on PRKCG, the active components of DFD were screened using molecular docking and molecular dynamics simulations. A total of 127 components in DFD with high GI were obtained (**Supplementary Table 5**), which proved that these components are active components of DFD in the treatment of UC. The molecular docking results of 127 DFD active components with PRKCG are shown in the **Supplementary Table 5**, all these active components can bind to PRKCG, and the binding energy of 39 components was ≤7.0 kcal·mol-1, indicating that these components have strong binding activity with PRKCG (21). The five active ingredients with the strongest binding activity were selected for visualization (**Figure 8**). We further performed molecular dynamics simulations for 100 ns of the five active components with the strongest binding activity, the results further confirmed the stability of DFD active components binding to PRKCG (**Supplementary Figure 2** and **3**). Taken together, these findings provide a strong rationale for further clarifying PRKCG as a key target of DFD in the treatment of UC.

### 3.8 DFD ameliorated UC mainly by affecting the PKCγ/ERK/NF-κB signaling pathway

**Figure 9:**
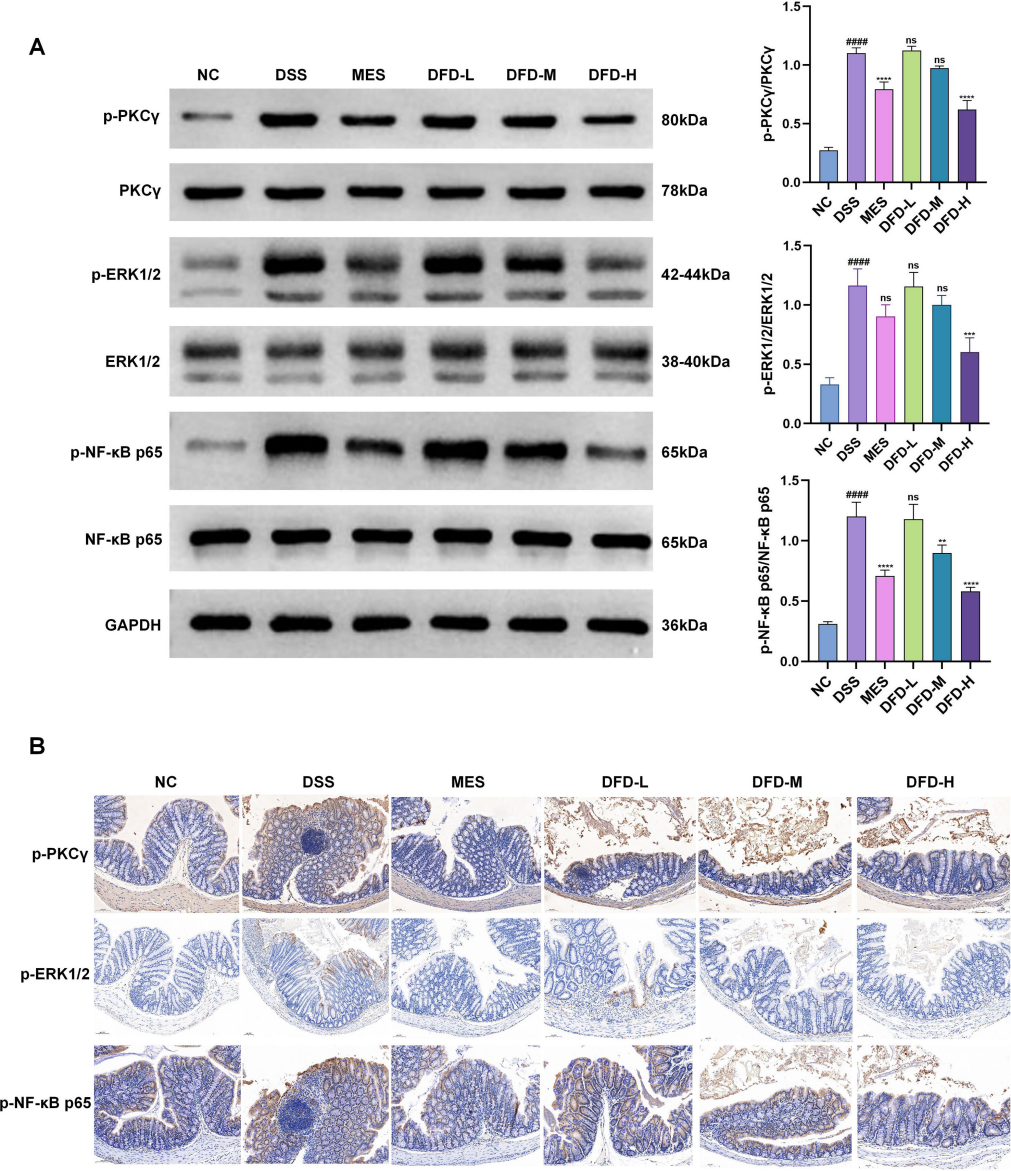
DFD ameliorates UC mainly by affecting the PKCγ/ERK/NF-κB signaling pathway. **(A)** The protein levels of p-PKCγ, PKCγ, p-ERK1/2, ERK1/2, p-NF-κB p65, and NF-κB p65 in colon tissue were detected by western blotting. **(B)** The expression of p-PKCγ, p-ERK and p-NF-κB in colon tissue was detected by IHC. Scale bar, 100 µm. Data are presented as mean ± SD (n = 3). ^####^*P* < 0.0001 *vs*. the NC group; ^**^P < 0.01, ^***^P < 0.001 ^****^P < 0.0001 *vs*. the DSS group; ns: not significant.

Chromosome 19q13.4.2 contains the PRKCG gene, which encodes for the PKCγ protein. PKCγ belongs to the PKC protein kinase family that governs many signal transduction and cellular processes (22). PKCγ regulates ERK, which in turn affects NF-κB and causes inflammation (23). Therefore, DFD is likely to improve UC by affecting the PKCγ/ERK/NF-κB signaling pathway. We conducted western blotting to detect changes in PKCγ, ERK, and NF-κB protein levels. As shown in **Figure 9A**, in comparison with the NC group, the DSS group showed an increase in PKCγ, ERK, and NF-κB phosphorylation levels in the colon (*P* < 0.0001), and these levels were markedly reduced following DFD treatment (*P* < 0.0001). Moreover, similar results were observed by IHC (**Figure 9B**). These results suggest that DFD improves UC largely by blocking the PKCγ/ERK/NF-κB signaling pathway.

## 4 Discussion

UC is marked by recurrent intestinal inflammation, impaired barrier function, and dysbiosis of the gut flora (24). Although a variety of drugs have been developed, UC still presents as a recurrent and incurable condition and poses a chief threat to public health worldwide (25). Therefore, new and effective therapeutic strategies are urgently needed. DFD (patent number: No. 201510162437. X) is a modified TCM patent formula on the basis of Lizhong decoction. In the context of UC, the substantial efficacy of DFD has been elucidated in our previous studies (10–12), although the mechanisms responsible for its activity have not been systematically delineated. In our study, we explored the function of DFD and the potential regulatory processes in a UC mouse model. To achieve this goal, we integrated transcriptome sequencing, untargeted metabolomics sequencing, and performed 16S rRNA sequencing to reveal the mechanisms activated by DFD treatment in UC.

UC pathogenesis is complex, with no consensus on its underlying (26). Studies have demonstrated that the dysbiosis of gut flora is implicated in UC pathogenesis (27), and researchers have documented a central effect of the gut flora in UC (28, 29). In UC, disorders of the intestinal flora often manifest as a decrease in overall diversity, lessened commensal useful bacteria, and harmful bacteria proliferation (28). To date, the significant therapeutic effects achieved by microbiota transplantation therapy, probiotics in the treatment of UC precisely illustrate this point (30, 31). In the present study, UC mice exhibited reduced bacterial diversity, which was restored by DFD treatment, especially with a significant regulatory effect on beta diversity. In addition, this study revealed that the microbiota structure of UC mice changed, as shown by an increased abundance of harmful bacteria as well as a lessened abundance of beneficial bacteria. However, following DFD treatment, the structures of beneficial and harmful bacteria in UC mice improved and tended toward normal. For example, at the phylum level, *Firmicutes* was reduced in patients with UC (32), and this study revealed a similar phenomenon in UC mice, as shown by a decline in the relative abundance of *Firmicutes*, which was reversed by DFD treatment. Moreover, *Proteobacteria* are harmful bacteria that have been unveiled to be tightly involved in IBD (6). Here, DSS disrupted the intestinal microbiota and increased the abundance of *Proteobacteria*. In contrast, DFD reduced *Proteobacteria* abundance. At the genus level, DSS increased *Enterococcus* abundance (a pathogenic bacterium), which was reversed by DFD intervention. The above findings are consistent with previous reports (6, 33). Hence, one of the most important roles for DFD in treating UC is the restoration of the equilibrium of the gut flora.

**Figure 10:**
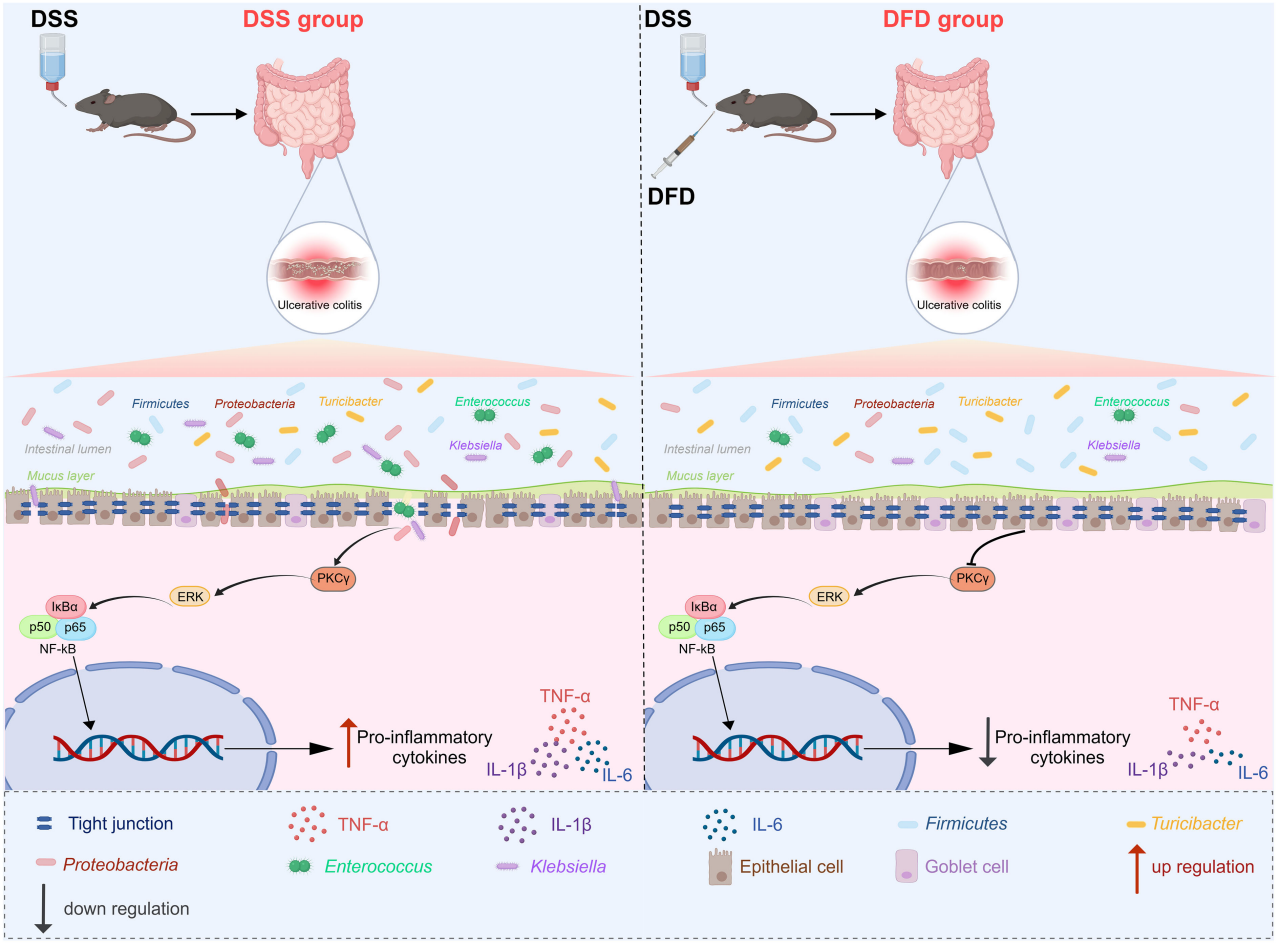
The mechanism of DFD in the treatment of UC.

An integrated multi-omics analysis is an effective method to study the pathogenesis of complex diseases and to explore the role of drugs in the treatment of diseases (15). Therefore, for the sake of thoroughly investigating the key mechanism of DFD, an integrated strategy encompassing metabolomics and transcriptomics was selected. First, the integrated analysis of metabolomics and transcriptomics revealed that 249 DEGs were correlated with 76 DEMs. These DEGs and DEMs were enriched in 17 signaling pathways, among which PRKCG was one of the targets with the largest number of involved pathways. Analysis of the relative expression of PRKCG mRNA in the transcriptome revealed raised PRKCG mRNA levels in UC colon tissues, but PRKCG mRNA was decreased subsequent to DFD treatment. Furthermore, molecular docking and molecular dynamics simulations further confirmed that the active components of DFD could bind stably to PRKCG. Thus, PRKCG is likely to be a key target for DFD for the treatment of UC. Chromosome 19q13.4.2 contains the PRKCG gene, which codes for the PKCγ protein (a PKC family member) that governs many signal transduction and cellular processes (22, 34). PRKCG and its encoded protein PKCγ were first found to be expressed only in neurons (35) and was associated with nervous system diseases (36). Later, PRKCG and its encoded protein PKCγ were found to be expressed in different tumors (37), and associated with the progression of breast cancer, prostate cancer, and other tumors (34, 38). In recent years, studies have shown that PRKCG mRNA or PKCγ protein was increased in inflammation-related disorders such as sepsis (39) and osteoarthritis (40), indicating that PRKCG or PKCγ is likely related to inflammation. However, relevant studies of PRKCG or PKCγ in UC are lacking. In this study, qPCR verified that PRKCG mRNA increased after exposure to DSS but decreased following treatment with DFD, further indicating that PRKCG controls UC treatment by DFD. PKC is an important member of the MAPK signaling pathway, it can phosphorylate its downstream protein ERK (37, 41). MAPKs can act as upstream activators of NF-κB. After activation, NF-κB translocates to the nucleus and activates the transcription of proinflammatory cytokines (23). The excessive accumulation of proinflammatory cytokines can cause damage to the intestinal barrier (42), which manifests as mechanical barrier damage with lower occludin, ZO-1, and claudin-1 levels and increased claudin-2 levels, alongside mucus barrier damage with goblet cell loss, leading to UC. The PKC/ERK/NF-κB pathway is triggered in arthritis, and interfering with its signaling can effectively alleviate arthritis (23). In the present study, similar results were observed, such as the activation of PKCγ/ERK/NF-κB signaling in the colitis samples, which was inhibited by DFD treatment. In addition, DSS treatment lowered ZO-1, occludin, and claudin-1 mRNA and protein levels, raised IL-1β, TNF-α, and IL-6 serum levels, lessened the goblet cells in colon tissues, and raised claudin-2 levels; however, DFD reversed these modifications. Therefore, DFD is likely to block PKCγ/ERK/NF-κB signaling, attenuate proinflammatory factor secretion and then repair intestinal mucosal barrier injury for UC management (**Figure 10**).

This study confirmed that DFD, as a modified patent formula of the Lizhong decoction, has a significant therapeutic effect on UC at the macroscopic and microscopic levels such as clinical manifestations, pathological damage of colon tissue, intestinal mucosal barrier, and serum inflammatory factors. The therapeutic effect of DFD is dose-dependent, with high dose of DFD, the clinically equivalent dose, achieving the best effect in the treatment of UC. In our previous real-world study (12), the application of this clinical equivalent dose in the treatment of UC also demonstrated significant efficacy, indicating that the application of this dose is scientifically reasonable. In terms of mechanistic studies, previous studies have investigated the spectrum-effect relationship of Lizhong decoction in the treatment of UC and confirmed that it regulates the intestinal flora and improves the abnormal metabolic profile of UC (8, 43, 44). This study also uncovered this effect, which further supports the effectiveness of DFD in the treatment of UC. In addition, this study integrated transcriptomics and metabolomics strategies, and identified a new target of DFD in the treatment of UC, which was verified by molecular docking, molecular dynamics simulation and *in vivo* experiments. This study enriched the mechanism of action of Lizhong decoction in the treatment of UC and provided a scientific basis for the prescription optimization and clinical translation of Lizhong decoction. It also provided a new potential therapeutic target for UC.

Some limitations to this study should be acknowledged. First, by integrating transcriptomics and metabolomics, this study found that PRKCG promoted the pathogenesis of UC, and DFD had a regulatory effect on PRKCG. This finding was only verified in UC mice, but was not validated in patients with UC, which limits the clinical generalizability of this finding. Therefore, future clinical studies should be conducted, especially to study the changes of PRKCG in UC patients at different stages (including active and remission stages) and the regulatory effect of DFD on PRKCG. Second, although PRKCG was found to be a key target of DFD in the treatment of UC, the effects of DFD on PRKCG and its downstream molecules were only examined by qPCR, IHC, and western blotting. In the future, PRKCG knockdown and overexpression experiments in mouse or cell models should be performed to more fully illustrate the key role of PRKCG in the treatment of UC by DFD. Third, this study found that DFD restores the intestinal flora imbalance in UC, but whether the treatment of UC by DFD is mediated by intestinal flora has not been confirmed. Therefore, future studies are needed to verify the mediating role of the gut microbiota in DFD treatment of UC by conducting antibiotic treatment and fecal microbiota transplantation experiments. These studies will accurately allow to better understand the therapeutic mechanisms of DFD and will provide strong scientific support for the clinical optimization of UC treatment, and will lay the foundation for the development of TCM for the treatment of UC.

## 5 Conclusion

By integrating multi-omics technology, this study analyzed the therapeutic effects and potential mechanisms responsible for DFD treatment of UC at the level of intestinal microflora-metabolite-gene-phenotype. In UC mice, DFD has substantial anti-inflammatory effects, and its pharmacological mechanism correlates with reshaping intestinal flora structure, inhibiting the PKCγ/ERK/NF-κB signaling pathway, reducing proinflammatory factors, and thereby restoring the intestinal mucosal barrier. Overall, this study provides a new perspective for investigating novel therapeutic strategies for UC.

## Data Availability

The datasets presented in this study can be found in online repositories. Transcriptomics data has been uploaded to the NCBI database (SRA data: PRJNA1429838), Untargeted metabolomic data has been uploaded to the MetaboLights database (MTBLS13973), 16S rRNA gene sequencing data has been uploaded to the NCBI database (SRA data: PRJNA1427912).

## Supplementary material

Supplementary Table 1 Primers sequences of qPCR.

Supplementary Table 2 T3 column mobile phase gradient conditions.

Supplementary Table 3 AB TripleTOF 6600 mass spectrometry conditions.

Supplementary Table 4 Pearson correlation analysis between DEGs and DEMs.

Supplementary Table 5 127 components in DFD with high GI and the results of molecular docking.

Supplementary Figure 1 Representative images of immunofluorescence staining for ZO-1, occludin, claudin-1 and claudin-2. Scale bar, 100 µm. n = 3.

Supplementary Figure 2 The results of molecular dynamics simulation. **(A)** Root mean square deviation (RMSD). **(B)** Radius of gyration (Rg). **(C)** Root mean square fluctuation (RMSF). **(D)** Hydrogen Bonds. **(E)** Solvent-accessible surface area (SASA). **(F-J)** Free energy distribution, binding free energy and amino acid residue contribution plots of PRKCG-23-Hydroxytormentic acid **(F)**, PRKCG -Euscaphic acid **(G)**, PRKCG-Asiatic acid **(H)**, PRKCG-Ellagic acid **(I)**, PRKCG-Quillaic acid **(J)**.

Supplementary Figure 3 Structural changes of the 5 complexes at different time points. **(A)** PRKCG-23-Hydroxytormentic acid. **(B)** PRKCG -Euscaphic acid. **(C)** PRKCG-Asiatic acid. **(D)** PRKCG-Ellagic acid. **(E)** PRKCG-Quillaic acid. The colors red, green, blue, yellow, and orange, respectively, represent the structural comparisons of molecular dynamics simulations at 0, 25, 50, 75, and 100 ns.

Supplementary method The operational procedures of molecular dynamics simulation.
