# Supplementary material for: Elucidating the molecular mechanisms of Daifu decoction in ulcerative colitis treatment through a multi-omics framework and experimental verification
Source: Front Immunol. 2026 Mar 24;17:1780747. doi: 10.3389/fimmu.2026.1780747 (PMC13054662; doi:10.3389/fimmu.2026.1780747)
Supplement: Supplementary Table 1 — Primers sequences of qPCR. [file Table1.docx]

**The operational procedures of molecular dynamics simulation**

Employing Gromacs 2024.4 software, molecular dynamics simulations were conducted on the complexes of PRKCG with 23-Hydroxytormentic acid, Euscaphic acid, Asiatic acid, Ellagic acid, and Quillaic acid. For the protein, we selected the Amber14sb force field, while the Gaff2 force field was selected for the ligands. The TIP4P water model added solvent to the protein-ligand system, and a 1.2-nm periodic boundary formed a water box. We used the Particle Mesh Ewald method (PME) to compute long-range electrostatic interactions. The Monte Carlo ion placement method introduced appropriate sodium and chloride ions to neutralise the system’s charge. Before the formal simulation, we minimised and equilibrated the system energy using three steps: (1) Minimising each system’s energy by the steepest-descent algorithm with 50,000 steps (stopping when the maximum force <1000 kJ/mol). (2) Each system was pre-equilibrated with 50,000 steps of 2fs at a constant particle number, volume, and temperature (310K). (3) The entire system was pre-equilibrated with a constant particle number, pressure (one atmosphere pressure), and temperature (310K) for 50,000 steps at 2 fs step size. After system energy minimization and equilibration, molecular dynamics simulations were performed for 100ns without any constraints in a step time of 2 fs, while the structural coordinates were saved every 10 ps.
 Finally, we analyzed the root mean square deviation (RMSD), root mean square fluctuation (RMSF), the radius of gyration (Rg), the solvent-accessible surface area (SASA), the number of hydrogen bonds between complexes, the relative free energy energy distribution, and the structural changes of the 5 complexes at different time points (0, 25, 50, 75, and 100 ns). Moreover, we calculated the mean binding free energy of five small molecules and PRKCG using the MM/GBSA method.
