# Supplementary figures and images for: Elucidating the molecular mechanisms of Daifu decoction in ulcerative colitis treatment through a multi-omics framework and experimental verification
Source: Front Immunol. 2026 Mar 24;17:1780747. doi: 10.3389/fimmu.2026.1780747 (PMC13054662; doi:10.3389/fimmu.2026.1780747)

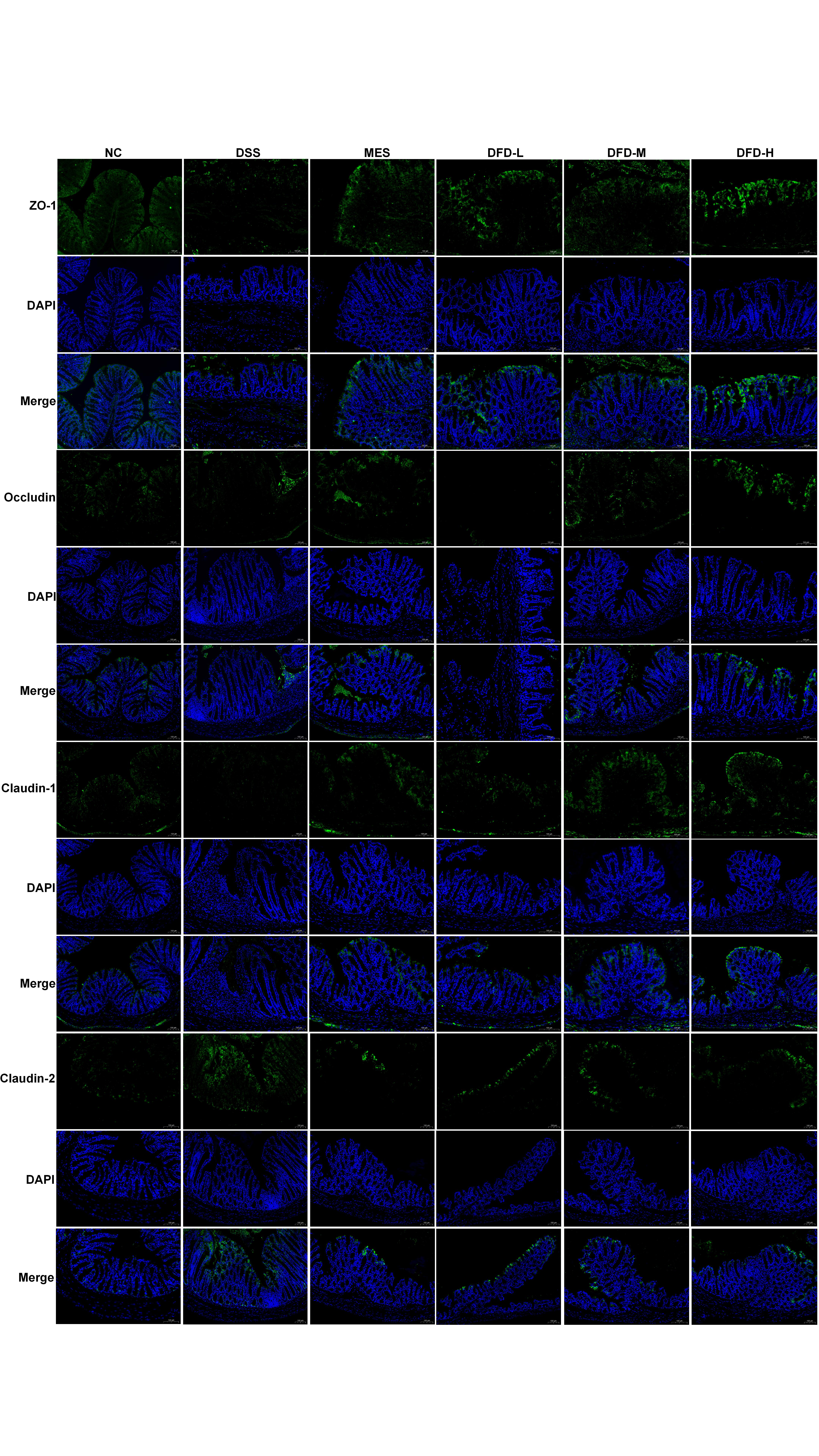

Supplement: Supplementary Figure 1 — Representative images of immunofluorescence staining for ZO-1, occludin, claudin-1 and claudin-2. Scale bar, 100 µm. n = 3. [file Image1.jpeg]

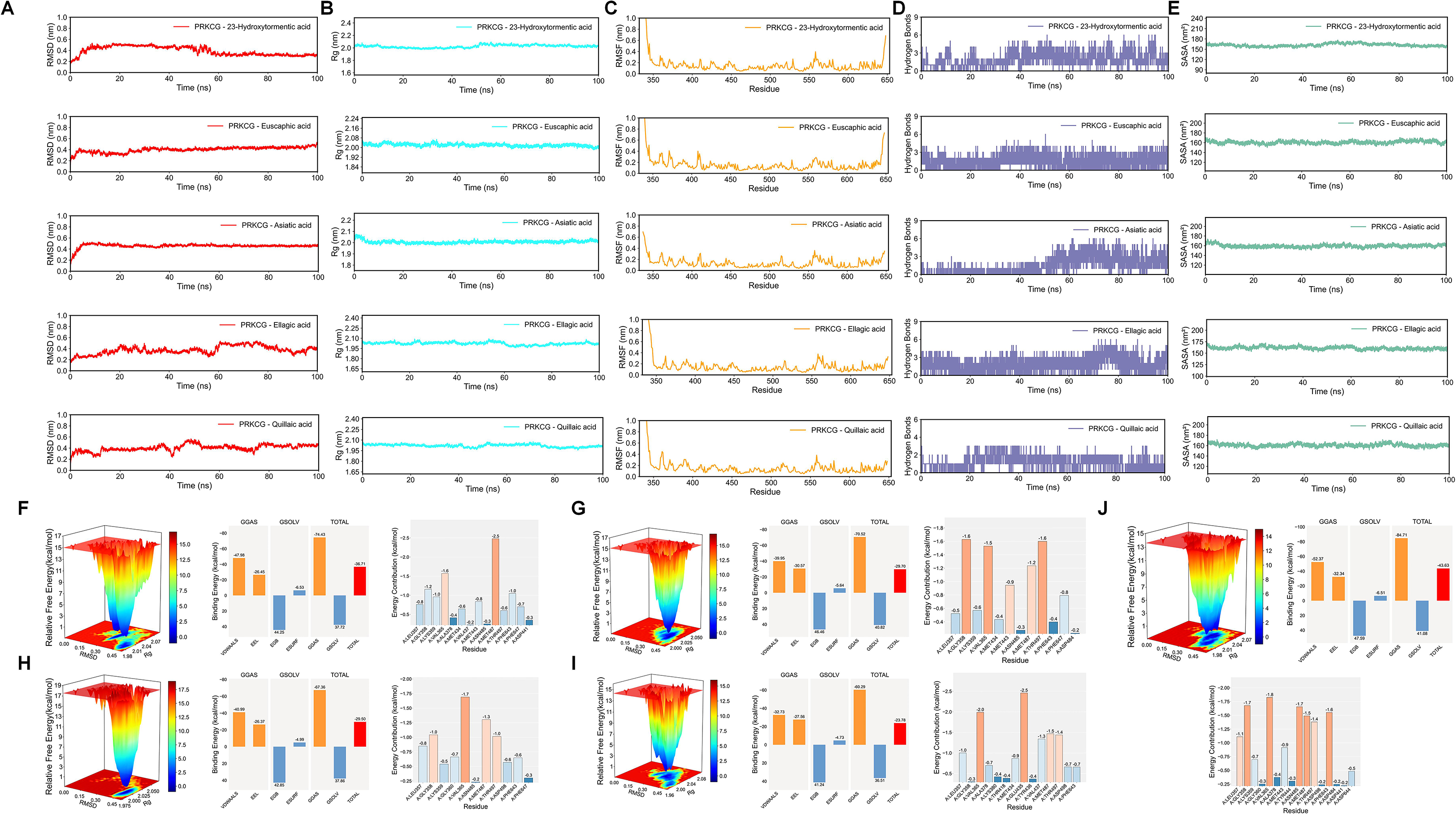

Supplement: Supplementary Figure 2 — The results of molecular dynamics simulation. (A) Root mean square deviation (RMSD). (B) Radius of gyration (Rg). (C) Root mean square fluctuation (RMSF). (D) Hydrogen Bonds. (E) Solvent-accessible surface area (SASA). (F-J) Free energy distribution, binding free energy and amino acid residue contribution plots of PRKCG-23-Hydroxytormentic acid (F), PRKCG -Euscaphic acid (G), PRKCG-Asiatic acid (H), PRKCG-Ellagic acid (I), PRKCG-Quillaic acid (J). [file Image2.jpg]

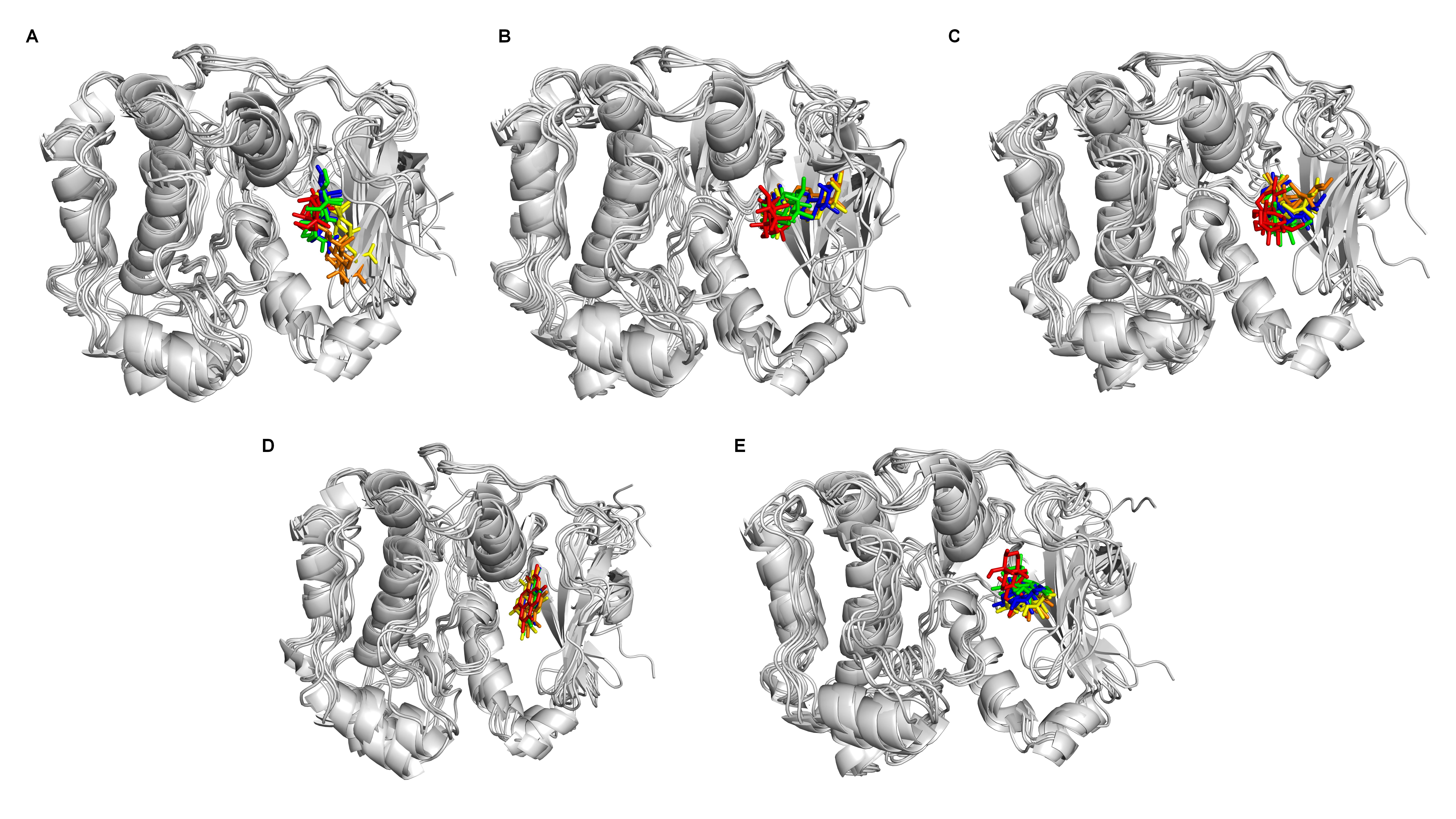

Supplement: Supplementary Figure 3 — Structural changes of the 5 complexes at different time points. (A) PRKCG-23-Hydroxytormentic acid. (B) PRKCG -Euscaphic acid. (C) PRKCG-Asiatic acid. (D) PRKCG-Ellagic acid. (E) PRKCG-Quillaic acid. The colors red, green, blue, yellow, and orange, respectively, represent the structural comparisons of molecular dynamics simulations at 0, 25, 50, 75, and 100 ns. [file Image3.jpeg]
